# Seed-Mediated Synthesis of Tunable-Aspect-Ratio Gold Nanorods for Near-Infrared Photoacoustic Imaging

**DOI:** 10.1186/s11671-018-2734-8

**Published:** 2018-10-04

**Authors:** Pengwei Li, Yiduo Wu, Dingding Li, Xiaoxiao Su, Cuixian Luo, Ying Wang, Jie Hu, Gang Li, Huabei Jiang, Wendong Zhang

**Affiliations:** 1 Micro-Nano System Research Center, College of Information and Computer, Taiyuan University of Technology, Taiyuan, 030024 Shanxi China; 2 College of Mechanics, Taiyuan University of Technology, Taiyuan, 030024 Shanxi China; 3 Biomedical Optics Laboratory, Department of Medical Engineering, College of Engineering, University of South Florida, Tampa, FL 33620 USA

**Keywords:** Gold nanorod, Tunable-aspect ratio, NIR window, Photoacoustic imaging

## Abstract

Tunable-aspect ratio gold nanorods have been synthesized by a modified seed-mediated synthesis method. Ascorbic acid was employed as a shape controller to induce anisotropic growth, which made the aspect ratio of the synthesized gold nanorods range from 8.5 to 15.6. These nanorods possess tunable longitudinal surface plasmon resonance absorption band, covering a broad near-infrared (NIR) range, from ~ 680 to 1100 nm. When modified with thiol-polyethylene glycol (SH-PEG), the synthesized Au nanorods showed excellent biocompatibility and stability, which foreshadowed the great potential of their NIR application as photoacoustic contrast agent. Due to their adjustable absorbance in the NIR, the synthesized Au nanorods could offer stronger contrast (3.1 times to the control group without contrast agent used) and higher signal-noise ratio values (SNR; 5.6 times to the control group) in photoacoustic imaging, both in vitro and in vivo experiments. Our work presented here not only added some novel Au-based photoacoustic contrast agents but also described a possibility of contrast agent preparation covering the whole biological NIR window.

## Background

One-dimensional (1D) nanostructures, such as nanowires, nanorods, nanotubes, and nanobelts, are especially interesting because they are not only novel basic building blocks for nanodevices, but also possess high geometrical aspect ratio producing anisotropic features for special applications [1–6]. Among of these 1D nanostructures, novel metal nanorods (NRs) have drawn increasing interests because of their shape-dependent surface plasmon resonance (SPR) band [7, 8], facile synthesis [9–11], favorable biocompatibility, and easy modification [12–14]. For example, Yeh et al. reported an Au nanorod (AuNR) in-shell structure smaller than 100 nm, which exhibits strong longitudinal absorbance at 600–900 nm and good applicability for the photo-induced therapies [8]. Wang et al. successfully constructed anisotropic AuNR helical superstructures with tailored chirality, by positioning the functionalized AuNR with DNA on the origami of the designed “X” pattern of the arrangement of DNA capturing strands [12].

In addition, improvements in synthesis and purification of AuNRs have enabled facile tuning of the longitudinal SPR band, by adjusting the length and hence aspect ratio [15–17], for specific application, like photoacoustic imaging (PAI) and photo-induced therapies [18–23], which need the longitudinal SPR of Au NRs to fall in the optical transparent window of biological tissue (first at 700–950 nm and second at 1000–1350 nm) [8, 18]. For instance, Huang and co-workers synthesized gold NRs with aspect ratio from 2.4 to 5.6, which displayed efficient cancer cell diagnostics and selective photothermal therapy [19]. Jokerst et al. developed gold NRs and silica-coated gold NRs with aspect ratio of about 3.5, which showed high PAI signal for ovarian cancer detection and mesenchymal stem cell imaging [20, 21]. Yang and co-workers reported magnetic gold nanorod/PNIPAAmMA for dual magnetic resonance PAI and targeted photothermal therapy [23]. Although many Au NR-based contrast agents have been developed, a facile, scalable synthesis of large and tunable-aspect ratio AuNRs and their absorption behavior-dependent PAI performance still remain challenges.

Herein, AuNRs with aspect ratio from 8.5 to 15.6 have been synthesized by using the modified seed-mediated growth method with the assistance of ascorbic acid. The AuNRs were demonstrated possessing with high biocompatibility and further reduced their cytotoxicity with SH-PEG modification. Benefiting from their large and tunable absorbance in the NIR region, the synthesized AuNRs could offer stronger contrast and higher signal-noise ratio (SNR) values in PAI, both in vitro and in vivo experiments. This facile method for building tunable-aspect ratio gold NRs may be utilized for fabricating contrast agent under any wavelength in the first NIR window.

## Experimental

### Synthesis of Gold Nanorods

Tunable-aspect ratio AuNRs were synthesized by a modified seed-mediated synthesis method [16, 17]. In a typical procedure, a volume of 10.3 mL of 0.025 M HAuCl_4_ (Sinopharm Chemical Reagent Co., Ltd., ≥ 99.9%) and 3.644 g of cetyl trimethylammonium bromide (CTAB) surfactant (Tianjin Guangfu Fine Chemical Research Institute, ≥ 99.0%) were first added to a beaker. Then, deionized water (18 MΩ) was added to bring the concentration of HAuCl_4_ to be 2.5 × 10^−3^ M, and CTAB of 0.1 M. 10 mL, 4.5 mL, 4.5 mL, and 45 mL of the above-mentioned solution were separately transferred into four flasks tagged as A, B, C, and D. Then, a volume of 350 μL, 0.01 M ice-cold NaBH_4_ (Sinopharm Chemical Reagent Co., Ltd., ≥ 98.0%) was added into flask A and stirred for 3 min. 0.4 mL solution of flask A and 25 μL 0.1 M L(+)-ascorbic acid (AA) (Tianjin Shentai Chemical Industry Co., Ltd., ≥ 99.7%) was transferred into flask B, stirred for another 3 min. And then, 0.4 mL solution of flask B and 25 μL of 0.1 M AA was added in flask C, stirred for 3 min again. Finally, 4 mL solution of flask C and 250 μL of 0.1 M AA was added in flask D, stirred for 5 s, and then left undisturbed in a water bath at 28 °C for 12 h. The top solution was removed carefully and the precipitate was centrifuged and washed several times with distilled water to make sure the excess CTAB was fully removed. Thus, the final products were signed as Au typical nanorods (AuTR).

Repeat the above process and just change the dosage of AA, and then Au NRs with aspect ratio from 8.5 to 15.6 could be developed. The details are as follows: the dosage of AA are (35 μL, 35 μL, 350 μL) for Au rod1, (30 μL, 30 μL, 300 μL) for Au rod2, (20 μL, 20 μL, 200 μL) for Au rod3, and (15 μL, 15 μL, 150 μL) for Au rod4.

### Surface Modification of AuNRs

First, 10 mg SH-PEG (Nanjing Pengsheng Biological Technology Co. Ltd) was dissolved in 1 mL deionized water and sonicated for 10 min. Then, the solution was treated with 50 mL 0.1 M NaBH_4_ solution under sonication for another 15 min to reduce the possible dimerized SH-PEG (PEG-S-S-PEG). Second, the cleaned-up Au NRs were dispersed into 10 mL deionized water and mixed to the above SH-PEG solution (10 ml), stirred for 5 min, and then placed undisturbedly for 5 h. Finally, the sample was centrifuged and washed with deionized water for further application.

### Characterization Methods

The morphology and structure of the synthesized AuNRs were identified by scanning electron microscopy (SEM; JEOL JSM-7001F) and transmission electron microscopy (TEM; JEOL 2100F, 200 kV). UV-vis absorbances of the various AuNRs were measured by spectrophotometer (Shimadzu, 3100 UV-vis-NIR). The photoacoustic signals were recorded by the unit rotation scanning photoacoustic detection system, which contains laser device (Surelite I-20, Continuum), optical parametric oscillator (OPO) (Surelite OPO Plus), non-focused ultrasonic transducer (PMUT) (V310-SU, Olympus, 5 Hz), motor step rotating table and its motor control box (MC) (M600, Beijing Zolix Instrument Co., Ltd.), preamplifier (5077PR, Olympus), PCI4732 data acquisition (DAQ) card, and so on.

### Cell Viability Experiments

All bio-experimental procedures were approved by IACUC committee at the Taiyuan University of Technology. And the experiments were carried out in accordance with the approved guidelines.

Hela cells were cultured in the standard cell medium recommended by American type culture collection (ATCC), at 37 °C under a 5% CO_2_ atmosphere. Cells seeded into 96-well plates were incubated with different concentrations of AuNR and AuNR-PEG for 24 h. Relative cell viabilities were determined by the standard methyl thiazolyl tetrazolium (MTT) assay and imaged under optical microscope.

### In Vitro and In Vivo PAI

Two grams of agar powder (Gene Company Ltd.) was dissolved in 100 mL deionized water and mixed well by glass bar in a beaker. The turbid liquid was heated to boiling in a microwave oven (Midea Group Limited by Share Ltd.). Then, the liquid was took out and stirred in water bath for 20 min at 60 °C, until the liquid became thick. Then, the viscous materials were poured into a 4.5 cm diameter cylindric mold, cooled, and solidified. Finally, the clotted agar was used as the phantom of biological tissue, due to their approximate absorbance to NIR lasers.

A 0.9-mm diameter glass capillary was implanted to the surface of the phantom to simulate blood vessel, which would be fulfilled with fresh ox blood, or blood mixed with various concentration of AuNR-PEG in specific experiment. The phantom was placed under water, and irradiated by 680-nm or 800-nm laser, at power density of 11 mJ/cm^2^.

Narcotize the mouse by isoflurane transiently, then 0.04 mL/10 g 10 wt% chloral hydrate was intraperitoneally injected to make the mouse anesthesia thoroughly. The mouse head was gently shaved of hair and smoothly smeared ultrasonic coupling agent (Boline Healthcare Ltd.). The wavelength of the laser was adjusted to 800 nm, and the mouse was placed under water. Then, the cerebral blood vessels of the mouse were imaged, before and after, and the contrast agents (1 nM, 0.1 mL/10 g) were intravenously (I.V) injected into the mouse. The laser was changed to 680 nm, and repeat the experiment above. Note: When the contrast agent was changed, the I.V injection should take at least 24 h later to let the residuum completely metabolized.

## Results and Discussions

**Fig. 1:**
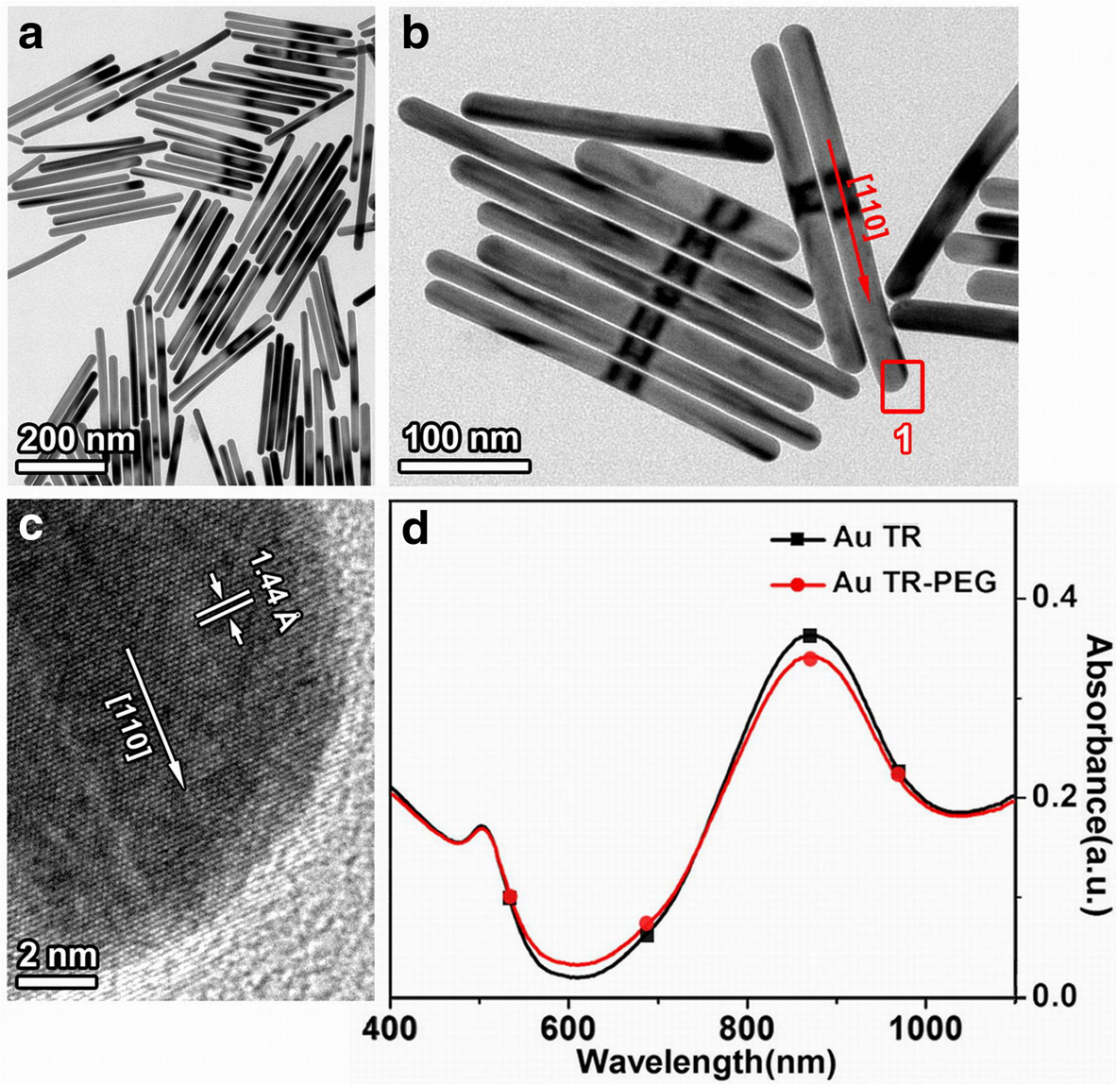
Typical morphology and structure of Au nanorod synthesized at 25 μL 0.1 M AA (AuTR): **a** Bright-field TEM image. **b** Amplification TEM image, of a single rod. **c** HRTEM image of a single rod in rectangular area “1” from panel **b**. **d** UV-vis absorption spectra of AuTR and AuTR-PEG

Typical morphology and structure of AuTR have been discussed systemically by TEM (Fig. 1). As shown in Fig. 1a, the synthesized AuTR (dosage of AA was 25 μL) are homogeneous in shape, with diameter of 22 ± 1.5 nm, length of 290 ± 13 nm, and the aspect ratio of around 13.2. Figure 1b shows a high-magnification TEM image of some representative AuTR. A high-resolution TEM (HRTEM) image of the end region of a single nanorod (“1” rectangular area in Fig. 1b) is shown in Fig. 1c. The result of which shows that lattice fringes perpendicular to the long axis of the nanorod can be discerned with d-spacings of 1.44 Å, corresponding to the (110) lattice plane. The nanorod grows along the [110] direction, as determined by the cubic structure of Au, from the analysis of selected area electron diffraction (SAED) pattern and HRTEM image [16, 17]. The UV-vis absorption spectra of AuTR in Fig. 1d demonstrate two absorption peaks, the characteristic peak at around 520 nm and longitudinal peak at about 900 nm. When functionalized with HS-PEG (the red line), the absorption band shows a slight decline (about 5%) on peak strength, but no obvious shift on peak position.

**Fig. 2:**
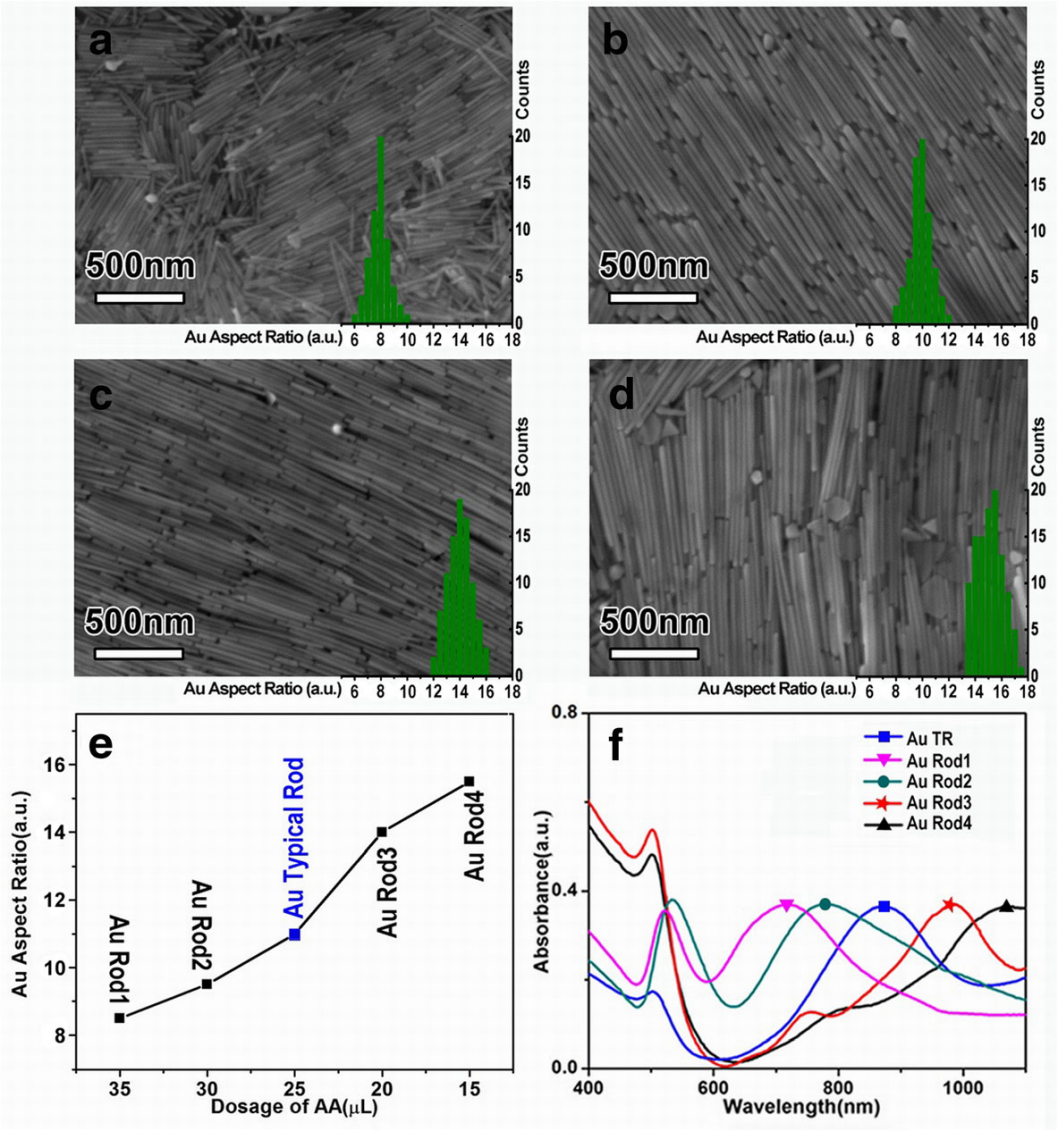
Morphology and aspect ratio statistics of AuNRs with different AA dosages: **a–d** SEM and histogram, **a** Rod1, **b** Rod2, **c** Rod3, and **d** Rod4. **e** The line chart of AA dosage corresponding to the aspect ratio. **f** The UV-vis absorption spectra of different AuNRs

It is well-known that the kinetic control of the monomer concentration and the crystal growth rate are the key factors to manipulate the particle size as well as the material shape initiated from the anisotropic growth [24, 25]. Thus, in this work, concentration-dependent experiments were performed to explore the influence of AA on the anisotropic growth of Au NRs. When the usage of AA is 35 μL (0.1 M), the aspect ratio of the synthesized AuNRs is about 8.5 ± 0.6 (Fig. 2a, about 50 individual AuNRs were randomly selected for the mathematical statistics of aspect ratio). Cutting down the dosage of AA from 35 to 15 μL, the aspect ratio of the AuNRs increases from 8.5 to 15.6 (Fig. 2a–d). Generally, AA is often used as reducing agent to reduce light yellow Au^3+^ to Au^+^ and could not induce the formation of Au^0^ nanoparticles [26, 27]. However, in our experiment, the aspect ratio of the synthesized AuNRs varies with the concentration of AA. It is suspected that AA is not only acting as a reducing agent, but also playing the role of capping agent to assist regulating the anisotropic growth of AuNRs in our experiment [28–30]. With the reducing of AA concentration in reaction system, Au^+^ ions are bound to accelerate their release and induce the rapid growth along the longitudinal axis of Au nanorod (Fig. 2e). Figure 2f shows the UV-vis absorption spectra of all the samples. With the aspect ratio increases from 8.5 to 15.6, the strong longitudinal SPR absorption band of the AuNRs red shifts from ~ 680 to 1100 nm, covering a broad NIR range (Fig. 2f), indicating their great potential for biomedical applications [31, 32].

**Fig. 3:**
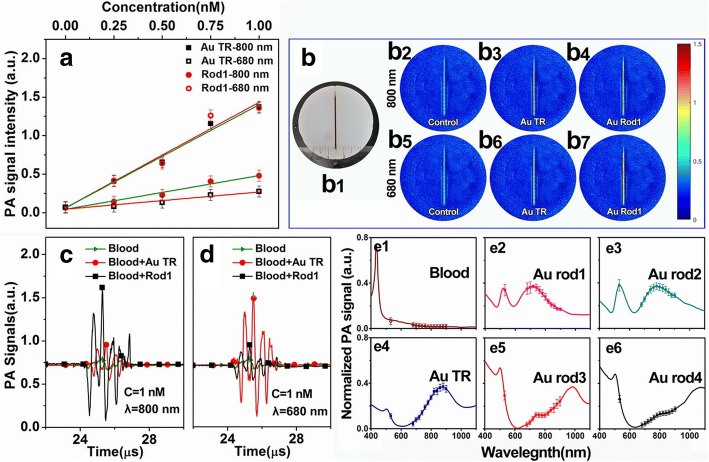
In vitro photoacoustic properties of AuTR and AuNRs: **a** concentration-dependent photoacoustic signal intensity of AuTR and Au rod1 irradiated by 800- and 680-nm laser, respectively, **b** PAI of glass capillary fulfill with blood balance mixed with 1 nM AuTR or Au rod1 irradiated by 800- and 680-nm laser, **c**, **d** the comparison of photoacoustic signal amplitude between pure blood and blood balance mixed with 1 nM AuTR or Au rod1 irradiated by 800- and 680-nm laser, **e1**–**e6** comparison of absorption spectra (solid line) of five kinds of synthesized AuNRs and fresh ox blood obtained from multi-wavelength photoacoustic signals amplitude (data points)

In vitro photoacoustic properties of AuNRs have been presented in Fig. 3. The photoacoustic (PA) amplitudes of AuNRs functionalized with HS-PEG were determined at a series of concentrations of the optical components from 0.25 to 1.0 nM (Fig. 3a), which showed good linear relations. AuTR provides large enhancement in PA signal irradiated by 800-nm laser and Au rod1 at 680 nm. When the laser wavelength was adjusted inadequately (e.g., AuTR at 680 nm and Au rod1 at 800 nm), the intensity of PA signal was sharply weakened. Figure 3b shows the PA images of glass capillaries fulfilling with fresh ox blood, or blood balance mixed with 1 nM AuTR and Au rod1. The results of which indicate that b3 (AuTR at 800 nm) and b7 (Au rod1 at 680 nm) have better imaging effect. Apparently, appropriate contrast agent could provide stronger absorption in PAI, resulting in the higher resolution of PA images. Figure 3c, d, presents the quantitative comparisons of photoacoustic signals between pure blood, and blood mixed with AuTR and rod1. The results of which show that photoacoustic signal amplitude of blood mixed with AuTR is 2.3-fold higher than pure fresh ox blood at 800 nm, and Au rod1 group is 2.1-fold higher at 680 nm. The large enhancements appear at the positions of their longitudinal absorption peaks. In other words, the absorption behavior of the AuNRs dominates their PAI performance.

The photoacoustic and optical spectra of five kinds of AuNRs and blood are shown in Fig. 3e1–e6. The multi-wavelength photoacoustic signal spectra were obtained by collecting amplitudes of photoacoustic signals at different wavelength (from 680 to 900 nm) lasers, with 1 nM aqueous solution was fulfilled in glass capillary tubes. Clearly, the graphs indicate a good agreement between the photoacoustic signal spectra and the optical spectra of the AuNRs. These results plain indicate the feasibility of applying AuNRs in PAI under suitable wavelength lasers and quantitatively give the photoacoustic effect of AuNRs at various wavelengths from 680 to 900 nm.

**Fig. 4:**
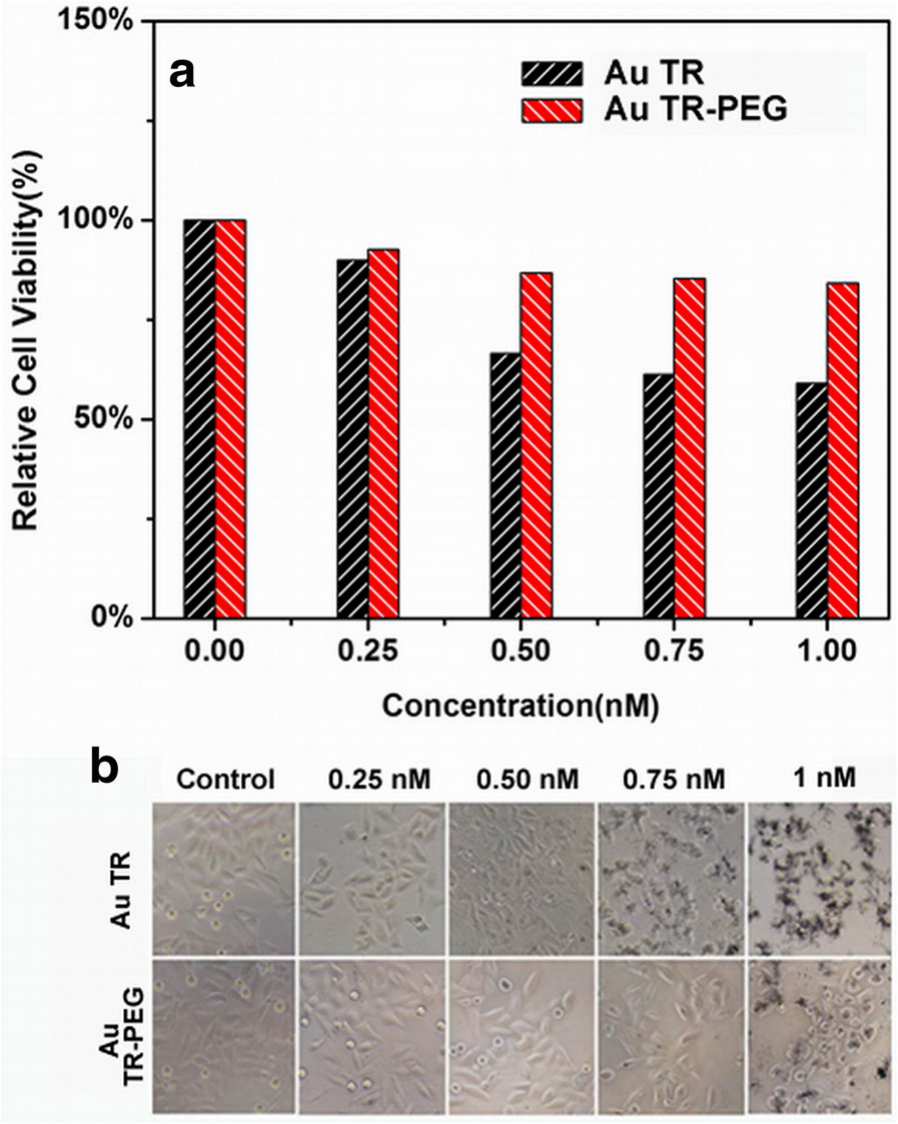
Relative viabilities of Hela cells after being incubated with various concentrations of AuTR with and without PEG modified within 24 h: **a** histogram of relative cell viabilities and **b** optical microscopy images of Hela cells

To exam the biotoxicity of AuNR on active targeting, Hela cells were incubated with AuTR with concentrations of 0.25–1.0 nM. The standard MTT assay was carried out to determine the viability of the cells (Fig. 4a). The results of which confirm that the combination of AuTR-PEG induce the greatest cell survival rate (95.3% at 1 nM), comparing with other groups within 24 h. It suggests that AuNR-PEG possess low cell cytotoxicity and good biocompatibility [33, 34] and maybe a promising photoacoustic contrast agent. Though the pure AuTR did not have significant toxicity (cell viability can get 71.2% at 1 nM), the cell death appeared at the concentrations of 0.75 and 1.0 nM (Fig. 4b), indicating that low concentration of AuTR is more suitable for photoacoustic imaging, while high concentration could induce cell death [35, 36]. Hence, for consideration of both photoacoustic-enhanced efficacy and biotoxicity of AuNRs, the concentration of 1 nM was chosen as the suitable condition for in vivo PAI.

**Fig. 5:**
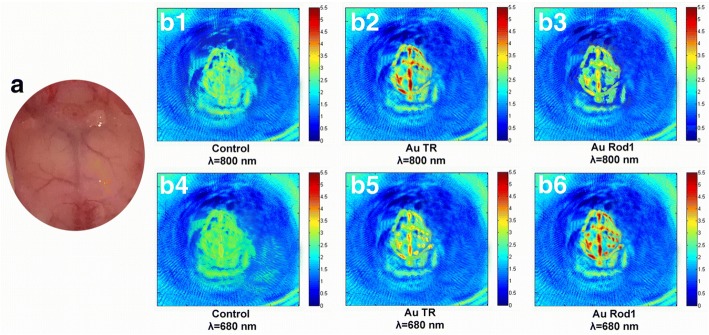
Photograph and PA images of mouse brain blood vessels: **a** photograph of mouse cerebrovascular, **b** PA images scheme of the mouse cerebral blood vessels before and after intravenous injection of AuTR or Au rod1, irradiated by 800- and 680-nm wavelength laser

Photoacoustic imaging is a non-invasive imaging modality offering an increased in vivo imaging depth and spatial resolution compared to other traditional optical imaging methods [37–40]. We found that AuNR-PEG with high NIR absorbance could be used as a great contrasting agent in photoacoustic imaging (Fig. 5). Figure 5a shows the photograph of the brain blood vessels of a mouse which is selected as the in vivo PAI specimen. Figure 5b1–b6 presents the photoacoustic images of mouse brain blood vessels for the specimen with and without AuNR-PEG additives, at 800- and 680-nm wavelength lasers, respectively. The results of which show that before the AuNR-PEG injection, there are only roughly shapes of main brain blood vessel in the control group PA images (Fig. 5b1, b4), and some branch vessels are blended in the background and hard to distinguish, no matter which wavelength of laser is used. When the contrast agent (AuTR-PEG and Au rod1-PEG) were injected in, the quality of the PA images have been greatly improved, and some disappeared fine branch vessels of the brain (in control group) emerge up clearly, especially the images of AuTR-PEG captured at 800 nm and Au rod1-PEG at 680 nm.

Photoacoustic images of Fig. 5b1–b6 were also quantitatively analyzed (Table 1) from aspects of contrast and signal-noise ratio (SNR). The average contrast of the whole image corresponding to every pixel was calculated from ten points, which were randomly selected on the same position of mouse brain blood vessels. Average contrast of the control group images is 1.113 in Fig. 5b1 and 1.076 in Fig. 5b4. After being injected with AuNR-PEG, the quality of all the images is enhanced at different degrees. In AuTR/800 nm group, the aorta is clear to observe (Fig. 5b2), and the average contrast can reach up to 3.451, 3.1 times to the control group. In a parallel comparison to Au rod1/800 nm group (Fig. 5b3), the average contrast is only 1.514, 1.36 times to the control group. However, when the wavelength of the laser changed to 680 nm, the contrast of AuTR is only 1.925, much lower than that of Au rod1 (3.692, 3.6 times to the control group). The SNR of pictures in AuTR group have been optimized 5.6 times at 800 nm to control group, and the Au rod1 group have also been enhanced 5.7 times at 680 nm. These results are basically consistent with that in vitro, that is, the large improvements in image quality can be ascribed to their respective large longitudinal absorption peaks.

**Table 1:** Comparison of contrast and SNR in PA images from b1 to b6 in Fig. 5. Contrast here is a mean value of the images for mice cerebral blood vessels; SNR in the table here denotes signal-noise ratio, which is the analysis of the whole pictures

Name	Aspect ratio	Absorption peak	Laser	Num	Contrast	SNR
Control	–	–	800 nm	b1	1.113	0.245
			680 nm	b4	1.076	0.304
AuTR	13.2 ± 1.1	800 nm	800 nm	b2	3.451	1.378
			680 nm	b5	1.925	0.655
Au Rod1	8.5 ± 0.6	680 nm	800 nm	b3	1.514	0.419
			680 nm	b6	3.692	1.726

## Conclusions

By the assist of ascorbic acid, tunable-aspect ratio gold nanorods, ranging from 8.5 to 15.6, have been synthesized by a modified seed-mediated synthesis method. These gold nanorods could provide tunable absorption peaks from 680 to 1100 nm, covering the first biological NIR window. When modified with SH-PEG, the synthesized AuNRs show excellent biocompatibility and stability, which foreshadows the great potential of their near-infrared application as photoacoustic contrast agent. Both experiments in vitro and in vivo confirm that the synthesized tunable-aspect ratio AuNRs could offer stronger contrast and higher SNR values in PAI, under suitable wavelength lasers. This work provides a possible way to controllably synthesize the contrast agent under any wavelength in the first NIR window and used for visualizing diseases such as intracerebral hemorrhage and thrombus.

## Abbreviations

1D: One-dimensional
AA: l(+)-ascorbic acid
AuNR: Au nanorod
AuTR: Au typical nanorods
CTAB: Cetyl trimethylammonium bromide
DNA: Deoxyribonucleic acid
MTT: Methyl thiazolyl tetrazolium
NIR: Near-infrared
NRs: Nanorods
OPO: Optical parametric oscillator
PA: Photoacoustic
PAI: Photoacoustic imaging
SAED: Selected area electron diffraction
SEM: Scanning electron microscopy
SH-PEG: Thiol-polyethylene glycol
SNR: Signal-noise ratio
SPR: Surface plasmon resonance
TEM: Transmission electron microscopy
